# Influence of the Single Nucleotide Polymorphisms rs12252 and rs34481144 in *IFITM3* on the Antibody Response after Vaccination against COVID-19

**DOI:** 10.3390/vaccines11071257

**Published:** 2023-07-19

**Authors:** Ieva Čiučiulkaitė, Winfried Siffert, Carina Elsner, Ulf Dittmer, Marc Wichert, Bernd Wagner, Lothar Volbracht, Frank Mosel, Birte Möhlendick

**Affiliations:** 1 Institute of Pharmacogenetics, University Hospital Essen, University of Duisburg-Essen, 45147 Essen, Germanybirte.moehlendick@uk-essen.de (B.M.); 2 Institute for Virology, University Hospital Essen, University of Duisburg-Essen, 45147 Essen, Germany; 3 Department of Clinical Chemistry and Laboratory Medicine, University Hospital Essen, University of Duisburg-Essen, 45147 Essen, Germany; 4 Institute of Medical Microbiology, University Hospital Essen, University of Duisburg-Essen, 45147 Essen, Germany

**Keywords:** SARS-CoV-2, mRNA-1273, BNT162b2, IFITM3, rs12252, rs34481144, antibody titer against SARS-CoV-2 RBD

## Abstract

The COVID-19 mRNA vaccine is the first mRNA vaccine approved for human administration by both the U.S. Food and Drug Administration and the European Medicines Agency. Studies have shown that the immune response and the decay of immunity after vaccination with the COVID-19 vaccines are variable within a population. Host genetic factors probably contribute to this variability. In this study, we investigated the effect of the single-nucleotide polymorphisms rs12252 and rs34481144 in the *interferon-induced transmembrane protein (IFITM) 3* gene on the humoral immune response after vaccination against COVID-19 with mRNA vaccines. Blood samples were collected from 1893 healthcare workers and medical students at multiple time points post-vaccination and antibody titers against the SARS-CoV-2 S1 protein receptor binding domain were determined at all time points. All participants were genotyped for the rs34481144 and rs12252 polymorphisms in the *IFITM3* gene. After the second and third vaccinations, antibody titer levels increased at one month and decreased at six months (*p* < 0.0001) and were higher after the booster vaccination than after the basic immunization (*p* < 0.0001). Participants vaccinated with mRNA-1273 had a higher humoral immune response than participants vaccinated with BNT162b2. rs12252 had no effect on the antibody response. In contrast, carriers of the GG genotype in rs34481144 vaccinated with BNT162b2 had a lower humoral immune response compared to A allele carriers, which reached statistical significance on the day of the second vaccination (*p* = 0.03) and one month after the second vaccination (*p* = 0.04). Further studies on the influence of rs12252 and rs34481144 on the humoral immune response after vaccination against COVID-19 are needed.

## 1. Introduction

In 2020, the novel severe acute respiratory syndrome coronavirus type 2 (SARS-CoV-2) caused a global pandemic resulting in 677 million cases and 6.9 million deaths worldwide (March 2023) [1,2]. Such a massive number of cases and deaths became a challenge not only to the health care system, but also to the socio-economic system [3,4].

Vaccines are a successful and cost-effective tool to protect individuals and populations against various pathogens and infectious diseases through herd immunity [5]. Several vaccines were approved for vaccination against COVID-19 in late 2020 and early 2021 [6]. Studies have shown that the immune response and the decay of immunity after vaccination with the COVID-19 vaccines are variable within a population. Factors that have been studied and shown to contribute to this variability include age, sex, pre-existing conditions, and choice of COVID-19 vaccine [7,8,9,10,11,12,13,14,15]. In addition, host genetic factors such as variants in the guanine nucleotide-binding protein β-3 subunit, the 3’ regulatory region 1 of the immunoglobulin heavy chain, or the human leukocyte antigen system also contribute to this variability [16,17,18,19].

*Interferon-induced transmembrane protein (IFITM) 3* is a membrane-associated protein that has antiviral activity by blocking the entry of the virus into the cell. The antiviral role of IFITM3 was discovered while screening for factors that could modulate influenza A virus infection. IFITM3 localizes on late endosomes and lysosomes and is able to restrict several viruses, including the influenza A virus, the Ebola virus, the hepatitis C virus, the dengue virus, and the severe acute respiratory syndrome coronavirus type 1 [20,21,22]. Further study demonstrated that IFITM3 also prevents the entry of SARS-CoV-2 into the cell [23].

The single-nucleotide polymorphism (SNP) rs12252 in the *IFITM3* gene has been reported to affect human susceptibility to influenza A and severity of influenza A infection as well as humoral immune response following influenza A vaccination [20,24,25,26]. The C allele of rs12252 has been associated with an *IFITM3* variant lacking 21 amino acids. The absence of these 21 amino acids causes IFITM3 to translocate from endosomal compartments to the cellular periphery and alters the antiviral activity of IFITM3 [27,28,29]. In the case of SARS-CoV-2, Xu et al. demonstrated that this truncated IFITM3 variant was not able to restrict SARS-CoV-2 infection, which was previously shown for influenza A as well [23,28]. Furthermore, Zhang et al. revealed that patients with severe influenza A infection were significantly more likely to have the CC genotype. The East Asian population is particularly affected by this polymorphism, as the minor allele frequency (MAF) of the C allele is significantly higher in this population [26,30]. In addition, Lei et al. showed that carriers of the rs12252 CC genotype had a lower humoral immune response after vaccination against the influenza A virus. In the same study, Lei et al. also demonstrated, that *IFITM3*-deficient mice had a lower antibody response after vaccination against influenza A as well as a decreased number of activated germinal center B cells and a defective germinal center-to-plasma/memory transition, which presented in a decreased number of plasmablasts and an increased number of memory B cells [20]. This finding may explain the lower humoral response in carriers of the rs12252 CC genotype after influenza A vaccination.

Another SNP in the *IFITM3* gene, rs34481144, has also been associated with the severity of influenza A infection. The A allele of this SNP increases the risk of severe influenza A infection. The SNP rs34481144 is located in the promoter region of the *IFITM3* gene and has been shown to affect *IFITM3* expression and transcription. The A allele was associated with lower *IFITM3* mRNA expression and reduced promoter activity as well as decreased binding of interferon regulatory factor 3, which plays a role in activating innate immune responses and controlling host responses to an infection, and increased binding of the inhibitory factor CTCF in the promoter region of *IFITM3* [31].

Further studies also found an association between *IFITM3* and SARS-CoV-2 infection. First, IFITM3 is upregulated in COVID-19 patients [32,33]. Second, as mentioned above, IFITM3 has the ability to restrict SARS-CoV-2 infection and prevent SARS-CoV-2 from entering the cell [23].

In addition, both SNPs in the *IFITM3* gene were associated with an increased risk of SARS-CoV-2 infection. Cuesta-Llavona et al. found a significantly higher frequency of carriers of the rs34481144 A allele and the rs12252 C allele in patients with COVID-19, and controls had a significantly higher frequency of the rs34481144 GG and rs12252 TT genotypes [34]. Further studies associated the C allele or the CC genotype of rs12252 with the risk of acquiring SARS-CoV-2 infection, severe SARS-CoV-2 infection and higher COVID-19 mortality [23,35,36,37]. Moreover, levels of antibodies against the receptor binding domain (RBD) and frequency of neutralizing antibodies were lower in COVID-19 patients with the CC genotype [23].

The COVID-19 mRNA vaccine is the first mRNA vaccine approved for human administration by both the U.S. Food and Drug Administration and the European Medicines Agency [38]. As mentioned above, both SNPs in *IFITM3* were associated with influenza A and SARS-CoV-2 infection. Furthermore, rs12252 also had an influence on the humoral immune response after vaccination against influenza A or by COVID-19 infection. With regard to vaccination against COVID-19, the impact of these SNPs in the gene *IFITM3* on the humoral immune response following immunization remains unknown. Therefore, we aimed to determine the association between rs34481144 and rs12252 SNPs in the *IFITM3* gene and humoral immune response after vaccination against COVID-19 with mRNA vaccines.

## 2. Materials and Methods

### 2.1. Study Group

For this study, 1893 healthcare workers and medical students were recruited from the University Hospital Essen (Essen, Germany). All participants were vaccinated with the COVID-19 vaccines BNT162b2 (Comirnaty^®^/COVID-19 vaccine from BioNTech/Pfizer, Mainz, Germany) or mRNA-1273 (Spikevax^®^/COVID-19 vaccine from Moderna Biotech, Madrid, Spain) and had no history of COVID-19.

For the basic immunization, participants were vaccinated twice with either BNT162b2 or mRNA-1273. By the third or the first booster immunization, we had four vaccination regimens: (i) three vaccinations with mRNA-1273, (ii) two vaccinations with mRNA-1273 and one vaccination with BNT162b2, (iii) three vaccinations with BNT162b2, (iv) two vaccinations with BNT162b2 and one vaccination with mRNA-1273. The booster vaccination was administered at least six months after the last vaccination.

We had no influence on the choice of vaccination regimen. The vaccinations were performed according to the vaccination protocols valid and the vaccines available in Germany at the time of the study.

The study was reviewed and approved by the Ethics Committee of the Medical Faculty of the University of Duisburg-Essen (21-10005-BO). All participants gave written informed consent to participate in this study.

### 2.2. Study Design

**Figure 1:**
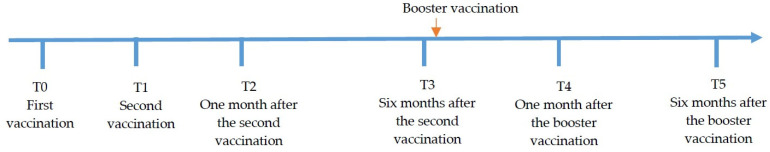
Schematic representation of study design. T0 is the day of the first vaccination, T1 is the day of the second vaccination, T2 is the time point one month after the second vaccination, T3 is the time point six months after the second vaccination, T4 is the time point one month after the booster vaccination, and T5 is the time point six months after the booster vaccination. The time interval between T0 and T1 depended on the chosen vaccine.

Blood samples were collected from the participants at six time points. The first blood sample was collected on the day of the first vaccination (T0). Participants who had a positive antibody titer or self-reported a previous COVID-19 infection at this time point were excluded from the study. T0 was also not included in the analyses because all participants enrolled in this study had negative antibody titers at this time point. The second blood sample was taken on the day of the second vaccination (T1). The time interval between T0 and T1 depended on the chosen vaccine. The interval varied between three and six weeks for the basic immunization with BNT162b2 and between four and six weeks for the basic immunization with mRNA-1273, based on the recommendations of the German standing vaccination commission (STIKO). Additional samples were collected one and six months after the second (T2 and T3, respectively) and third (T4 and T5, respectively) vaccination. All time points are shown schematically in Figure 1. A time period of +/− two weeks was allowed at the time points T2-T5. Antibody titers against SARS-CoV-2 S1 protein RBD and nucleocapsid protein were measured at each time point. The genotypes of the rs34481144 and rs12252 polymorphisms in the *IFITM3* gene were determined in all participants.

### 2.3. Genotyping of IFITM3

Genomic DNA was extracted from 200 µL EDTA-blood using the QIAamp^®^ DNA Blood Mini Kit (Qiagen, Hilden, Germany). The polymerase chain reaction (PCR) was performed with 2 µL genomic DNA and 16 µL *Taq* DNA-Polymerase 2x Master Mix Red (Ampliqon, Odense, Denmark) under the following conditions: Initial denaturation at 95 °C for 5 min; 35 cycles of denaturation at 90 °C for 30 s, annealing at 60 °C for 30 s and extension at 72 °C for 30 s each; final extension at 72 °C for 10 min (forward primer: 5′ ATG TGG ATC ACG GTG GAC G 3′; reverse primer 3′ AGG AAT TTG TTC CGC CCT CA 5′). PCR amplicons were purified using the ExS-Pure™ Enzymatic PCR Purification Kit (NimaGen, Nijmegen, The Netherlands). Cycle sequencing was then performed using the BrilliantDye™ Terminator (v3.1) Cycle Sequencing Kit (NimaGen, Nijmegen, Netherlands). Secondary purification of PCR amplicons was performed using the iX-Pure™ DyeTerminator Cleanup Kit, Resin Based (NimaGen, Nijmegen, Netherlands). All steps were performed according to the manufacturer’s standard protocols. Genotyping of all samples was performed via Sanger sequencing using the SeqStudio™ Genetic Analyzer according to the manufacturer’s instructions (Applied Biosystems™, Darmstadt, Germany).

### 2.4. Detection of Antibodies against SARS-CoV-2 Spike Protein

SARS-CoV-2 RBD antibody titers were evaluated using SARS-CoV-2 S1 RBD IgG/sCOVG tests with Siemens Atellica^®^ IM System (Siemens Healthcare GmbH, Erlangen, Germany) according to the manufacturer’s instructions. Antibody concentrations were reported in binding antibody units per milliliter (BAU/mL) as recommended by the World Health Organization. The detection limit for positivity was 21.8 BAU/mL, as recommended by the manufacturer.

### 2.5. Detection of Antibodies against SARS-CoV-2 Nucleocapsid Protein

SARS-CoV-2 nucleocapsid protein antibody titers were evaluated to exclude participants with previous SARS-CoV-2 infection. The Architect i2000SR CoV-2 IgG assay (Abbott Diagnostics, Abbott Park, IL, USA) was used according to the manufacturer’s instructions. Results with an index ≥ 1.4 were considered evidence of previous infection. SARS-CoV-2 nucleocapsid protein antibody titers were evaluated at each time point. Participants who had positive SARS-CoV-2 nucleocapsid protein antibody titers at any time point and/or self-reported previous COVID-19 were excluded from further analysis.

### 2.6. Statistical Analysis

Statistical analyses were performed using Graph Pad Prism 7 (Graph Pad Software, San Diego, CA, USA) and IBM SPSS Statistics 27 (IBM Software, Ehningen, Germany). Comparisons between more than two groups were performed using the Kruskal–Wallis test and Dunn’s multiple comparison test. Comparisons between two groups were performed using the Mann–Whitney test. Categorical data sets were evaluated using Pearson’s Χ^2^ test. Hardy–Weinberg equilibrium (HWE) was calculated using Pearson’s Χ^2^ goodness of fit test, and samples were deemed to deviate from HWE at a significance level of *p* < 0.05. The influence of rs34481144 and rs12252 in the *IFITM3* gene on antibody response was determined in homozygous (AA vs. aa), heterozygous (Aa vs. aa and Aa vs. AA), dominant (AA vs. Aa + aa), and recessive (aa vs. Aa + AA) models. Genotype TT of rs12252 and GG of rs34481144 corresponded to AA, and the genotypes of rs12252 CC and rs34481144 AA correspond to aa. *p*-values are given as two-sided, and values < 0.05 were considered significant.

## 3. Results

**Figure 2:**
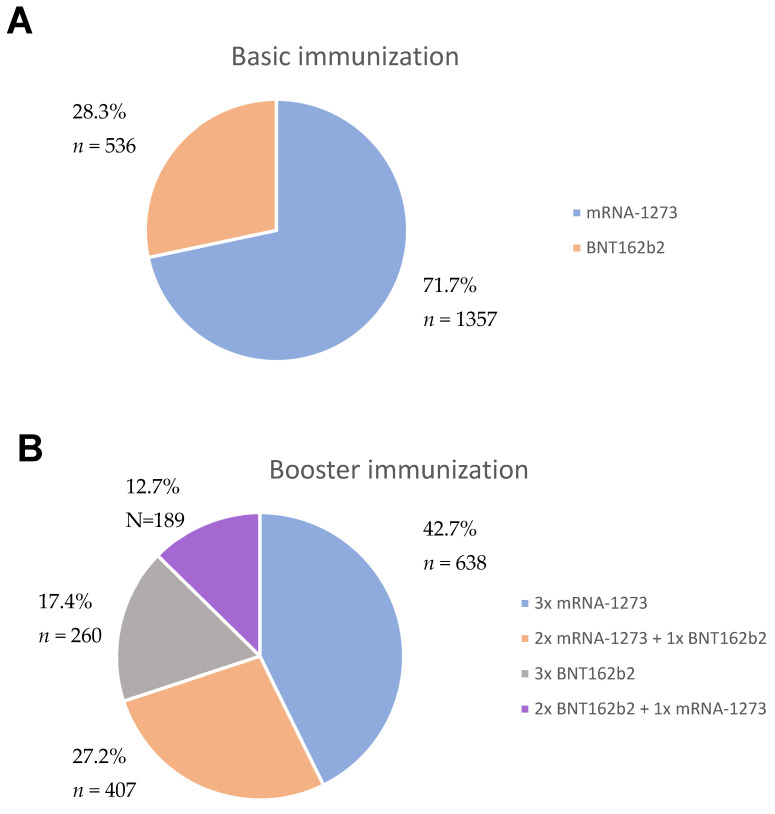
Distribution of mRNA vaccines by basic immunization and by booster vaccination among participants. Panel (**A**) shows the distribution of vaccines by basic immunization and panel (**B**) shows the distribution by booster vaccination.

In the study group of 1893 participants, 71.9% (*n* = 1361) were female and 28.1% (*n* = 532) were male. The median age of the study group was 35 years (range 18–72), and the median body mass index (BMI) was 24.2 kg/m^2^ (range 15.6–56.8). More participants received basic immunization and booster vaccination with mRNA-1273 than with BNT162b2 (Figure 2).

**Figure 3:**
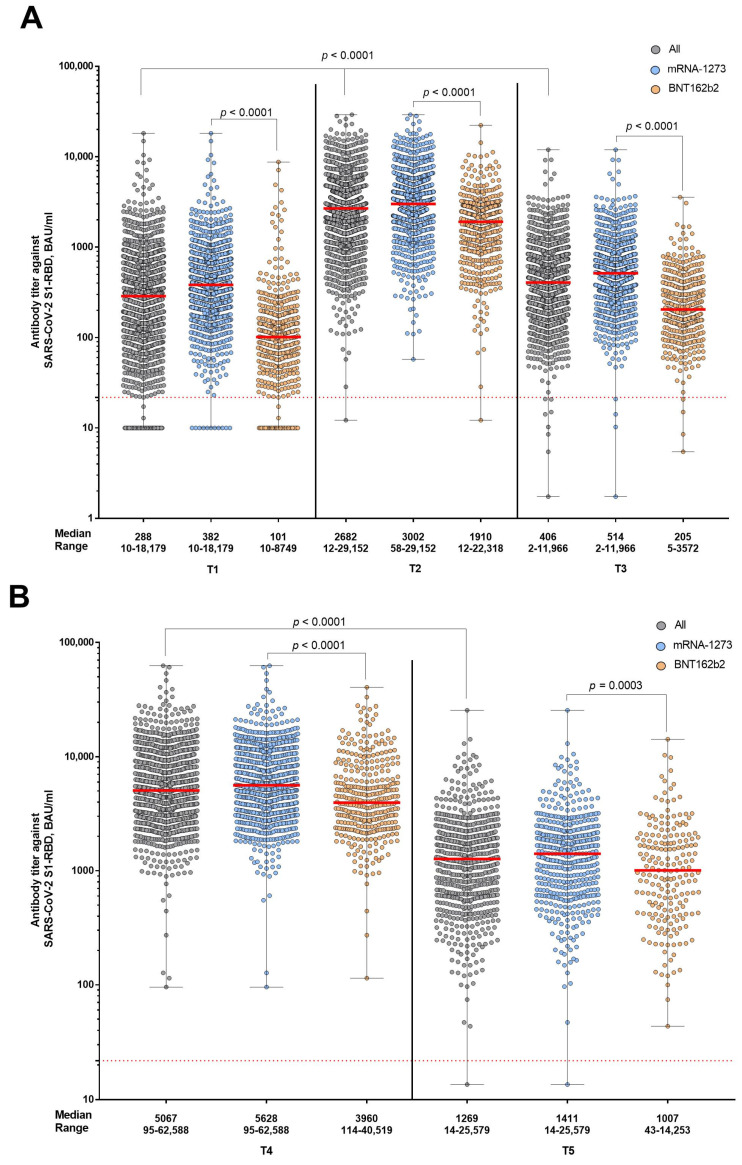
Kinetics of the humoral immune response in the main study group and in the subgroups of participants vaccinated twice with mRNA-1273 and twice with BNT162b2 as the basic immunization. Panel (**A**) shows the time points on the day of the second vaccination (T1) as well as at one month (T2) and six months (T3) after the second vaccination, respectively. Panel (**B**) shows the time points one (T4) and six (T5) months after the booster vaccination. Antibody titer levels are expressed in binding antibody units per milliliter (BAU/mL). The red dashed line indicates the cut-off for positivity (21.8 BAU/mL).

**Figure 4:**
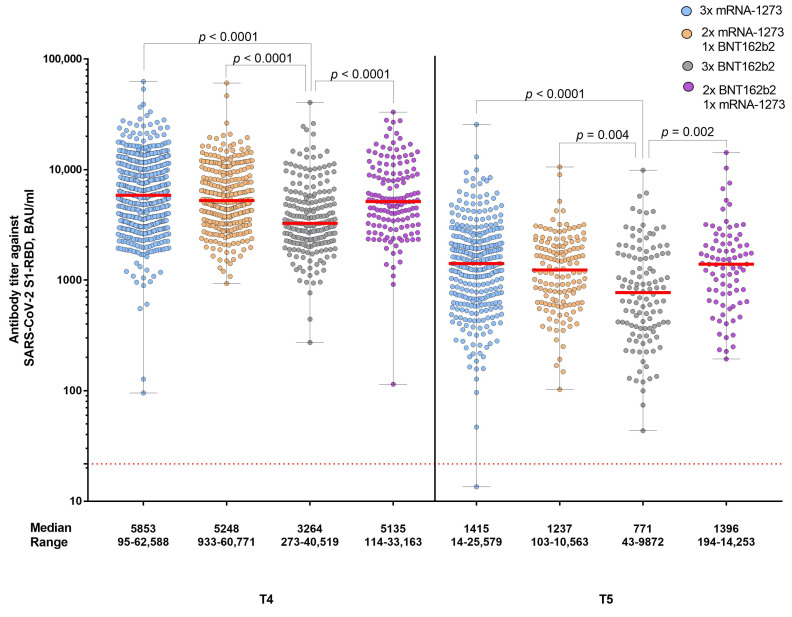
Humoral immune response after the first booster vaccination. The study group was divided into four vaccination regimens: (i) three vaccinations with mRNA-1273, (ii) two vaccinations with mRNA-1273, and one vaccination with BNT162b2; (iii) three vaccinations with BNT162b2; (iv) two vaccinations with BNT162b2 and one vaccination with mRNA-1273. Antibody titer levels are expressed as BAU/mL. The red dashed line indicates the cut-off for positivity (21.8 BAU/mL). T4 indicates the time point one month after the booster vaccination, and T5 indicates the time point six months after the booster vaccination.

After the second and third vaccinations, antibody titer levels increased at one month and decreased at six months (*p* < 0.0001) (Figure 3). At time point T4, antibody titer levels were higher than at time point T2 and at time point T5, higher than at time point T3 (*p* < 0.0001) (Figure 3). Participants who received basic immunization with mRNA-1273 or at least one dose of mRNA-1273 for the booster vaccination had higher antibody titers than those who had received vaccination with BNT162b2 (Figure 3 and Figure 4).

The influence of sex, age, and BMI on the humoral immune response after vaccination against COVID-19 is summarized in Table 1. Female participants had a better humoral immune response after the first vaccination, while the opposite trend, a stronger immune response in males, was observed at time points T2 and T3. Younger participants had higher antibody titer levels until the first booster dose. After the booster vaccination, the effect of age and sex considerably diminished. A BMI below 30 kg/m^2^ was associated with a better humoral immune response at T1 and T3. One month after the booster vaccination, the opposite trend was observed, with a higher BMI being associated with a better humoral immune response.

For *IFITM3* rs12252, 90.8% (*n* = 1715) of the participants had the TT genotype, 8.0% (*n* = 151) had the CT genotype, and 1.2% (*n* = 23) had the CC genotype. For *IFITM3* rs34481144, 28.9% (*n* = 546) of the participants had the GG genotype, 49.4% (*n* = 934) had the AG genotype, and 21.7% (*n* = 409) had the AA genotype.

Genotypes for *IFITM3* rs12252 were not consistent with HWE, and the minor allele frequency (MAF) was 0.05. Genotypes for *IFITM3* rs34481144 were compatible with HWE, and the MAF was 0.46.

Both polymorphisms rs12252 and rs34481144 in *IFITM3* had no effect on antibody response at any of the time points after vaccination against COVID-19 in the entire study group. As the immune response was significantly different between participants vaccinated with mRNA-1273 and with BNT162b2, we determined the influence of the two polymorphisms separately according to the vaccine used for basic immunization.

**Figure 5:**
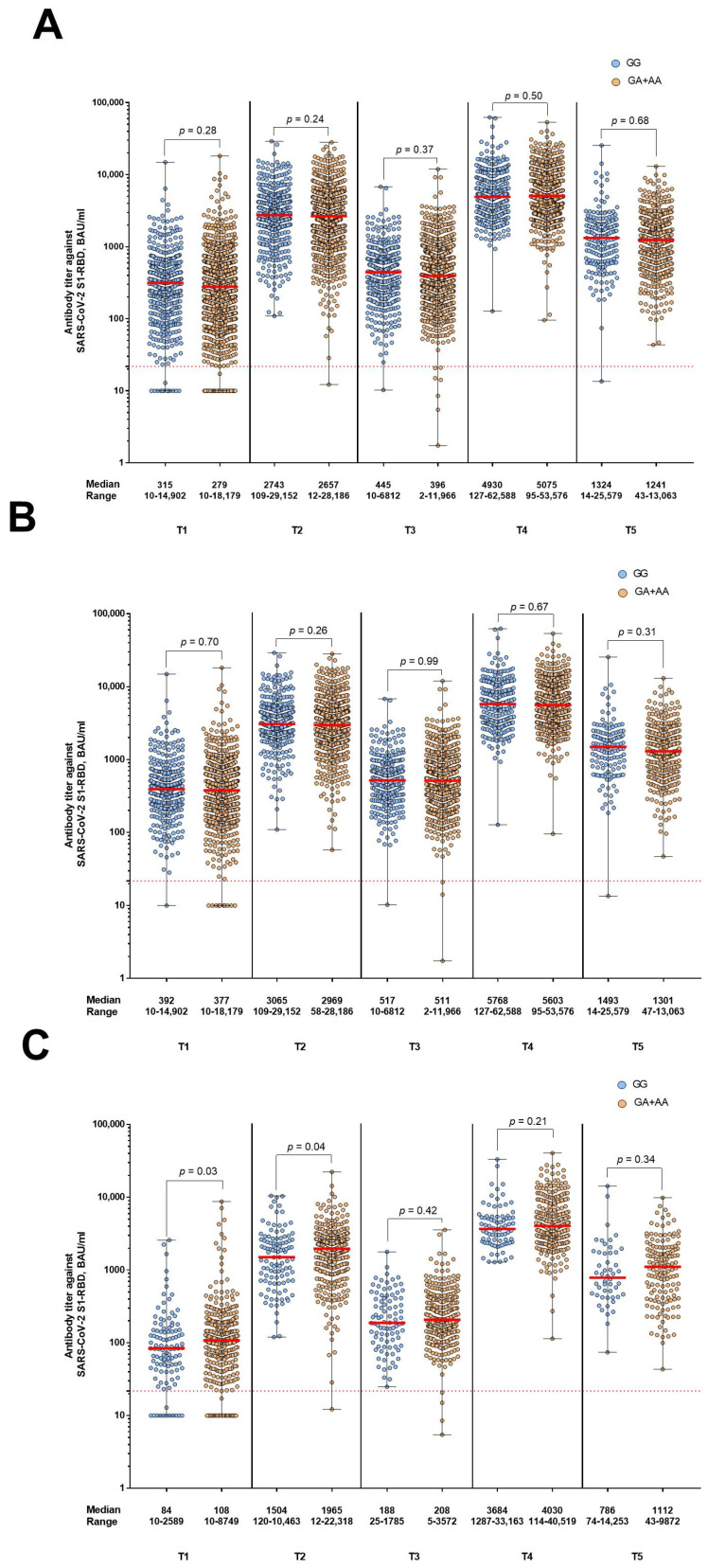
Association between rs34481144 polymorphism in the dominant model and humoral immune response at all time points in the main study group (**A**) and in the subgroups of participants vaccinated twice with mRNA-1273 (**B**) and participants vaccinated twice with BNT162b2 (**C**) as basic immunization. Antibody titer levels are expressed in BAU/mL. The red dashed line indicates the cut-off for positivity (21.8 BAU/mL). T1 is the day of the second vaccination, T2 is the time point one month after the second vaccination, T3 is the time point six months after the second vaccination, T4 is the time point one month after the booster vaccination, and T5 is the time point six months after the booster vaccination.

The *IFITM3* rs12252 genotypes and alleles had no effect on antibody titer levels after vaccination against COVID-19 with either mRNA-1273 or BNT162b2 as basic immunization at any time point. *IFITM3* rs34481144 had also no effect on antibody titer levels after vaccination with mRNA-1273 as basic immunization. Participants who received two doses of BNT162b2 as basic immunization and were carriers of the GG genotype had a trend toward lower antibody titer levels at all time points, reaching statistical significance at T1 and T2 (Figure 5).

To exclude the possibility that these significant differences in humoral immune response in BNT162b2 vaccine subgroup at time points T1 and T2 were caused by confounders, we examined whether there were differences in age and BMI or a different distribution of age groups (18–30 years, 31–50 years, 51–72 years), BMI groups (BMI < 30.0 and BMI ≥ 30.0), or sex between rs34481144 genotypes and between GG genotype and A allele carriers. We did not find any differences between rs34481144 genotypes or between the GG genotype and the A allele carriers.

## 4. Discussion

Vaccination against COVID-19 became an important measure to control the COVID-19 pandemic. In this study, we followed the humoral immune response after vaccination with mRNA vaccines against COVID-19 for more than one year. During this time, we observed the kinetics of the humoral immune response after basic immunization as well as after the first booster vaccination and investigated whether SNPs in the *IFITM3* gene influence the humoral immune response after vaccination against COVID-19 with mRNA vaccines.

The humoral immune response increased after one month and decreased six months after vaccination. Antibody titer levels were higher at the same time points after the first booster vaccination as after the basic immunization. In addition, we observed higher antibody titer levels in participants who received mRNA-1273 twice as a basic immunization or who received mRNA-1273 as a booster vaccination. Although similar kinetics of the humoral immune response have been observed in other studies, our study has some outstanding advantages, such as a large study group of 1893 participants, multiple testing points, and a long follow-up period in the same study group [10,11,13,39,40,41,42].

Furthermore, we examined the influence of factors such as age, sex, and BMI on the humoral immune response. The results regarding the influence of sex on the humoral immune response were inconsistent. Both sexes could be associated with a stronger immune response but at different time points. On the other hand, other studies on sex differences in the response to the COVID-19 vaccine have also reported conflicting results [7,13,14,15,40,43,44,45,46]. It is known that females more often have a better immune response after vaccination, and it seems that this tendency also applies to vaccination against COVID-19, although the data are not entirely consistent [47].

Younger age was associated with a better humoral immune response after basic immunization in our study group. These results are in agreement with several other studies [10,13,14,15,46,48,49]. After the booster vaccination, the influence of sex and age on the humoral immune response considerably diminished in our study group.

Finally, we investigated the influence of BMI on the humoral immune response after COVID-19 vaccination. Participants with a BMI below 30.0 kg/m^2^ tended to have higher antibody concentrations after the basic immunization. Interestingly, the opposite trend was observed one month after booster vaccination, where higher BMI was associated with higher antibody concentrations. Data from other studies on the influence of BMI are also inconsistent. Watanabe et al. reported that a higher BMI was associated with a lower antibody titer level, whereas Pellini et al. and Cangemi et al. found no association between BMI and the humoral immune response [15,48,50]. Regarding our results one month after the booster vaccination, the same pattern was observed by Holtkamp et al. In this study, professional firefighters with a higher BMI had a better humoral immune response. Holtkamp et al. speculated that a higher BMI does not necessarily reflect obesity and may be caused by higher muscle mass [49].

Although we had a representative study group of 1893 subjects, we observed differences in sex and BMI compared to the results of other studies and between different time points in our study. It seems that these common host factors play a role in the immune response after the vaccination against COVID-19, but the data, except for age, are not consistent and probably depend on the study group.

The genotypes of *IFITM3* rs12252 were not compatible with HWE, but the MAF of rs12252 in this study was similar to the MAF reported for the European population (MAF = 0.04) [30]. rs12252 has been shown to influence the humoral immune response after influenza A vaccination or SARS-CoV-2 infection, as well as the course of influenza A and COVID-19 [20,23,24,25,26,35,36,37] infection. In this study, we found no association between rs12252 and humoral immune response after vaccination against COVID-19 with mRNA vaccines. Our results correlate with those of Schönfelder et al., who also found no association between rs12252 and the risk of acquiring a SARS-CoV-2 infection or the severity of COVID-19 [51]. Studies associating rs12252 with humoral immune response or the course of influenza A and SARS-CoV-2 infection were often conducted in East Asian populations. Our study and the study by Schönfelder et al. were conducted in a German population. The MAF of rs12252 differs significantly between these two populations. In the East Asian population, the MAF of rs12252 is 0.53, which is much higher than in the European population and which may explain the differences in results between different studies [30]. Therefore, studies investigating the effect of rs12252 on the humoral immune response after vaccination against COVID-19 in the East Asian population would be a valuable and interesting contribution. This would help to better understand the role of rs12252 after vaccination against COVID-19. As far as the European population is concerned, rs12252 is probably not a factor that influences the immune response after vaccination against COVID-19.

The MAF of rs34481144 in this study was very similar to the MAF reported for the European population (MAF = 0.46) [30]. rs34481144 had no effect on the humoral immune response in the overall study group and in the subgroup of participants vaccinated with mRNA-1273. In the BNT162b2 vaccine subgroup, carriers of the rs34481144 GG genotype tended to have a lower humoral immune response, which reached statistical significance at two time points.

To date, there are no comprehensive studies on the influence of rs34481144 on the humoral immune response after vaccination against COVID-19. A few studies have investigated the influence of rs34481144 on the course and susceptibility of COVID-19, but their results were contradictory. Cuesta-Llavona et al. found an association of rs34481144 with susceptibility to COVID-19, whereas Schönfelder et al. found no association of rs34481144 with susceptibility to SARS-CoV-2 infection or the course of COVID-19. In the study by Cuesta-Llavona et al., the A allele was more common in patients than in healthy controls. Allen et al. also identified the A allele as a risk factor for severe influenza A infection which is associated with impaired expression and transcription of *IFITM3* [31,34,51].

In our study, the GG genotype was associated with a lower humoral immune response in the BNT162b2 vaccine subgroup. It is known that although both vaccines contain full-length mRNAs encoding spike proteins, there are a few differences between them, such as slight variations in the RNA sequences and different composition of the lipid nanoparticle vehicles. In addition, the two vaccines contain different amounts of mRNA per dose and different intervals between doses are recommended [52,53,54]. These variations in the composition and administration of the vaccines could be a possible explanation for different findings in BNT162b2 and mRNA-1273 vaccine subgroups.

Furthermore, to be sure that these results were not a fortuitous finding, we investigated whether other factors might have influenced the lower immune response in GG genotype carriers vaccinated with BNT162b2. We chose common factors such as age, BMI, and sex. However, in the group of BNT162b2 vaccinees, we found no differences in age, BMI, and sex between carriers of the different rs34481144 genotypes. Therefore, it appears that the rs34481144 GG genotype may be associated with a lower humoral immune response after BNT162b2 vaccination and possibly a higher susceptibility to SARS-CoV-2 infection, as Möhlendick et al. showed that breakthrough infections were associated with weaker antibody responses [55].

## 5. Conclusions

In this study, we investigated for the first time whether rs12252 and rs34481144 in the *IFITM3* gene influence the humoral immune response after vaccination against COVID-19 with mRNA vaccines. We had a comprehensive study group and a long follow-up period. BNT162b2 vaccinees carrying the GG genotype in rs34481144 had a lower humoral immune response. rs12252 had no effect on the humoral response, and it seems that this SNP is not a factor that could influence the immune response after vaccination against COVID-19 in the European population.

However, further studies are needed at this stage. First, our data should be replicated in an independent cohort. Second, further studies in populations with higher MAF of rs12252 would help to better understand the influence of rs12252 on the immune response after the vaccination. Finally, the molecular mechanisms by which the rs34481144 polymorphism influences the humoral immune response after the vaccination against COVID-19 are not clear and need to be investigated.

## Figures and Tables

**Table 1:** Association between sex, age, and body mass index (BMI) and the humoral immune response in the main study group and in the subgroups of participants vaccinated twice with mRNA-1273 and participants vaccinated twice with BNT162b2 as the basic immunization. Antibody titer levels are expressed in BAU/ml and in median (range). T1 is the day of the second vaccination, T2 is the time point one month after the second vaccination, T3 is the time point six months after the second vaccination, T4 is the time point one month after the booster vaccination, and T5 is the time point six months after the booster vaccination.

	T1	*p*-Value	T2	*p*-Value	T3	*p*-Value	T4	*p*-Value	T5		*p*-Value
**Sex**											
**All**											
female	323 (10-18179)	**<0.0001**	2642 (29-29152)	**0.03**	397 (9-11966)	**0.02**	4957 (127-62588)	0.15	1243 (14-25579)		0.32
male	223 (10-5465)		2797 (12-17888)		465 (2-9189)		5265 (95-60771)		1387 (96-13063)		
**mRNA-1273**											
female	413 (10-18179)	**<0.0001**	2895 (109-29152)	**0.0002**	482 (10-11966)	**<0.0001**	5419 (127-62588)	0.05	1407 (14-25579)		0.45
male	322 (10-5465)		3329 (58-17888)		652 (2-9189)		6259 (95-60771)		1482 (96-13063)		
**BNT162b2**											
female	109 (10-8749)	0.15	1891 (29-22318)	0.89	198 (9-3078)	0.46	3820 (273-40519)	0.49	926 (43-14253)		0.24
male	98 (10-2754)		1942 (12-11229)		221 (5-3572)		4239 (114-27694)		1287 (128-6725)		
**Age**											
**All**											
18–30	435 (10-14902)	**<0.0001**	3138 (135-28186)	**<0.0001**	597 (51-9149)	**<0.0001**	5131 (443-46487)	0.32	1267 (103-10563)		0.17
31–49	248 (10-18179)		2537 (12-29152)		350 (5-11966)		4911 (114-60771)		1206 (47-8473)		
50–72	133 (10-8749)		2369 (29-26264)		298 (2-6812)		5134 (95-62588)		1370 (14-25579)		
**mRNA-1273**											
18–30	474 (10-14902)	**<0.0001**	3384 (338-28186)	**<0.0001**	662 (51-9249)	**<0.0001**	5463 (933-46487)	0.08	1366 (103-10563)		**0.01**
31–49	345 (10-18179)		2890 (117-29152)		433 (21-11966)		5276 (606-60771)		1259 (47-8473)		
50–72	233 (10-2452)		2648 (58-26264)		424 (2-6812)		6058 (95-62588)		1552 (14-25579)		
**BNT162b2**											
18–30	177 (28-7137)	**<0.0001**	2481 (135-10463)	**0.0004**	357 (52-1785)	**<0.0001**	3944 (443-40519)	0.61	1052 (120-9872)		0.87
31–49	108 (10-4900)		1884 (12-22318)		196 (5-3572)		4208 (114-27694)		964 (74-7569)		
50–72	67 (10-8749)		1636 (29-8939)		167 (9-3078)		3792 (273-33163)		1000 (43-14253)		
**BMI**											
**All**											
<30	306 (10-18179)	**<0.0001**	2697 (12-29152)	0.29	425 (2-11966)	**0.001**	4918 (95-62588)	**0.005**	1240 (14-25579)		0.26
≥30.0	203 (10-8749)		2658 (68-28186)		324 (25-9249)		6157 (606-53576)		1522 (74-13063)		
**mRNA-1273**											
<30	392 (10-18179)	**0.005**	3061 (58-29152)	0.76	535 (2-11966)	**0.005**	5463 (95-62588)	**0.01**	1337 (14-25579)		0.11
≥30.0	332 (10-3093)		2865 (147-28186)		425 (57-9249)		6644 (606-53576)		1602 (215-13063)		
**BNT162b2**											
<30	109 (10-7137)	**0.02**	1945 (12-22318)	0.80	209 (5-3572)	0.28	3820 (114-40519)	0.12		978 (43-14253)	0.87
≥30.0	87 (10-8749)		1747 (68-8939)		183 (25-3078)		5252 (1243-23523)			1138 (74-4842)	

## Data Availability

The original contributions presented in the study are included in the article. Further inquiries can be directed to the corresponding author.
